# Mitochondrial and Nuclear Genes-Based Phylogeography of *Arvicanthis niloticus* (Murinae) and Sub-Saharan Open Habitats Pleistocene History

**DOI:** 10.1371/journal.pone.0077815

**Published:** 2013-11-01

**Authors:** Gauthier Dobigny, Caroline Tatard, Philippe Gauthier, Khalilou Ba, Jean-Marc Duplantier, Laurent Granjon, Gael J. Kergoat

**Affiliations:** 1 IRD, CBGP (IRD, Inra, CIRAD, Montpellier SupAgro), Campus de Baillarguet, Montferrier-sur-Lez, France; 2 Centre Régional Agrhymet, Rive Droite, Niamey, Niger; 3 Inra, CBGP (IRD, Inra, CIRAD, Montpellier SupAgro), Campus de Baillarguet, Montferrier-sur-Lez, France; 4 IRD, CBGP (IRD, Inra, CIRAD, Montpellier SupAgro), Dakar, Senegal; University of Florence, Italy

## Abstract

A phylogeographic study was conducted on the Nile grass rat, *Arvicanthis niloticus*, a rodent species that is tightly associated with open grasslands from the Sudano-Sahelian regions. Using one mitochondrial (cytochrome b) and one nuclear (intron 7 of Beta Fibrinogen) gene, robust patterns were retrieved that clearly show that (i) the species originated in East Africa concomitantly with expanding grasslands some 2 Ma, and (ii) four parapatric and genetically well-defined lineages differentiated essentially from East to West following Pleistocene bioclimatic cycles. This strongly points towards allopatric genetic divergence within savannah refuges during humid episodes, then dispersal during arid ones; secondary contact zones would have then stabilized around geographic barriers, namely, Niger River and Lake Chad basins. Our results pertinently add to those obtained for several other African rodent as well as non-rodent species that inhabit forests, humid zones, savannahs and deserts, all studies that now allow one to depict a more comprehensive picture of the Pleistocene history of the continent south of the Sahara. In particular, although their precise location remains to be determined, at least three Pleistocene refuges are identified within the West and Central African savannah biome.

## Introduction

Current climatic changes and their consequences on the evolution of biodiversity are a hotly debated topic with sometimes highly divergent predictive scenarios [1]–[6]. In most groups, long-term surveys are still lacking to provide robust predictions under various potential scenarios. In such a context, though at a very different scale, the reconstruction of past eco-climatic modifications may provide helpful pieces of information. In particular, when interpreted through available paleo-environmental frameworks, phylogeographic studies of habitat-specialist species can join the cortege of data that is necessary to draw the complete picture [7], [8]. Even more instructive is the comparison of phylogeographic patterns obtained in several species sharing the same biogeographic regions [9]–[11].

In Africa, a majority of phylogeographic investigations have been conducted on large mammals (see references below; review in [10]). Yet, the latter are now usually confined to protected areas where recent demographic and genetic effects may mask signatures of more ancient evolutionary events. In contrast, rodents appear as excellent alternative phylogeographic markers owing to their usually strong affinities to particular ecological niches and wide geographic distribution, their short generation time, their weak dispersal abilities as well as their small size that may render them highly sensible to geographic barriers such as mountains or large rivers, thus limiting admixture between allopatrically differentiated lineages. Altogether, these characteristics are expected to greatly facilitate detection of phylogeographic patterns. Moreover, most of rodent species being abundant and non-protected (when not pest organisms), they are relatively easy to sample, thus facilitating the gathering of datasets that are appropriate for phylogeographic/population studies. This may explain why all African species investigated so far led to strong phylogeographic signals, as well as remarkable congruence between sometimes highly divergent taxa. Indeed, data are now available for rodent species inhabiting arid zones (*Jaculus jaculus*: [12]), steppes, shrub and tree savannahs (*Lemniscomys striatus*: [13]; *Praomys daltoni*: [14]; *Mastomys erythroleucus*: [15], [16]; *Mastomys natalensis*: [17]), rocky habitats (*Acomys chudeaui*: [18]), humid areas (*Mastomys huberti*: [19]), forest-savannah mosaics [*Mus (Nannomys) minutoides*: [20] and forests (*Praomys rostratus* and *P. tullbergi*: [21]; *P. misonnei*: [22], [23]).

As part of this process, we here focused on the Nile grass rat, *Arvicanthis niloticus* (Muridae, Murinae). This species ranges all along the Southern limit of the Sahara desert, throughout the Sahelian and Sudanian regions, from the Atlantic to the Indian Oceans [24], [25]. As such, *A. niloticus* biogeographic evolution is expected to provide interesting insights on the effects of past eco-climatic changes on sub-Saharan steppes and savannahs. The genus *Arvicanthis* contains seven currently recognized species [24], all found south of the Sahara desert, from the Atlantic coast in Senegal to Ethiopia, and down south to Zambia, with the exceptions of populations along the Nile Valley in Egypt, south west of the Arabian Peninsula, and a mention from the Hoggar mountains, south east Algeria [24], [25]. Many of them represent sibling species whose boundaries and phylogenetic relationships have essentially been resolved thanks to cytogenetic and molecular studies [26]–[33]. Nevertheless, systematic investigations are still required, particularly for several East African taxa (see [32], and below) as well as other ambiguous taxonomic units (e.g. ANI-2 and ANI-4; see [34]).

The Nile grass rat *Arvicanthis niloticus* is a well-defined species characterized by a 2N = 62/number of autosomal arms (NFa) = 62–64 karyotype ([26], [28]–[30]). This species exhibits the widest distribution within the genus [24]. It is rather generalist and inhabits steppes, savannahs as well as humid zones and human-modified biotopes (e.g. villages, gardens, rice fields, sometimes within cities) of the Sahelian and Sudanian bioclimatic zones where it is considered a major agricultural pest [25]. *Arvicanthis niloticus* has also been shown to carry a number of pathogens, such as *Leishmania major* [35], *Borrelia* spp. [36], *Leptospira* spp. [37], *Rickettsia* spp. [38], *Schistosoma* spp. [39] or *Toxoplasma gondii* [40]. Yet, our understanding of evolutionary patterns in the Nile grass rat still relies on very scarce data, and no wide scale survey has been performed on this species to date. Previous cytogenetic studies have suggested the possible existence of two lineages, a West African one (ANI-1a, *sensu* [30]) and another Central and East African one (ANI-1b) whose karyotypes would differ by a pericentric inversion on pair 30 (NFa = 62 and 64, respectively; [30]). Recently, Abdel Rahman *et al.* [33] proposed some preliminary hypotheses about *A. niloticus* phylogeography and demogenetics using only mitochondrial DNA (mtDNA) sequence data. Unfortunately, their study was based on only 26 specimens, 23 of which were sampled in Sudan, making their results likely biased and uninformative at a wider scale.

In order to fill this gap, we performed a comprehensive phylogeographic survey of *A. niloticus* based on a sample that covers all the species range. In contrast with previous phylogeographic studies dealing with West and Central African rodents (see above), we used both a mitochondrial (cytochrome b) and a non-translated part of a nuclear (Beta Fibrinogen intron 7) coding genes to infer the evolutionary history of our species of interest. We also implemented more thorough time calibration analyses, by using independent set of constraints based either on fossil data or on secondary calibrations. Finally, our results are discussed in regards to those previously obtained for other rodent as well as non-rodent species in this part of Africa.

## Materials and Methods

### Sampling Effort

In total, 105 individuals of *A. niloticus* and two *A. ansorgei* individuals were trapped during various field surveys conducted in seven countries (Burkina-Faso, Cameroon, Chad, Mali, Niger, Mauritania and Senegal; Table 1). Organs or hind feet digits were conserved in ethanol until use in the laboratory. In addition, fibroblast cell pellets from one *A. niloticus* specimen from Egypt and two *A.* cf. *niloticus* individuals from Kenya were kindly provided by V. Volobouev (Muséum National d’Histoire Naturelle, Paris, France). Four ethanol-preserved samples of *A. niloticus* from Sudan (courtesy of E. Abdel Rahman, Natural Science Museum, Durban, South Africa) and one *A. ansorgei* from Burkina-Faso (courtesy of M. Deniau) were also available. All these specimens (115 in total) were sequenced for the mitochrondrial cytochrome b gene (cytb), while some representatives were selected among them for the intron 7 of the nuclear gene Beta Fibrinogen (Fib7) sequencing (see details below). In addition, 54 cytb sequences were downloaded from GenBank, among which 34 *A. niloticus* representatives (one from Egypt, one from Senegal, two from Niger, seven from Cameroon and 23 from Sudan), nine representatives of other *Arvicanthis* species as well as 11 representatives of other genera (*Aethomys, Desmomys, Golunda, Mylomys, Otomys, Pelomys*, and *Rhabdomys*). For the fib7 dataset we also used a sequence from a specimen of *Mastomys erythroleucus* that was trapped in Mali. Finally, one *Lemniscomys* cytb sequence was kindly provided by V. Nicolas (Museum National d’Histoire Naturelle, Paris, France). The rationale here (see also the *Phylogenetic and dating analyses* section for details) was: (i) to have a dense sampling of *Arvicanthis* species in order to properly enforce the corresponding fossil constraint; (ii) include species used in previous studies within Arvicanthini and Murinae [41], [42] in order to perform secondary calibrations. Associated voucher specimens are deposited in the collections of the Centre de Biologie pour la Gestion des Populations (CBGP, France), Muséum National d’Histoire Naturelle (MNHN, France) and Durban Natural Science Museum (DM); they can be recovered using numbers provided in Table 1.

**Table 1:** Samples used in this study, with the individual reference numbers of the different collections where they are registered.

Individuals and species	Origin	Lat.	Long.	cyt b	Fib7	Labels
*Arvicanthis niloticus*						
GenBank: AF004568.1*	Ethiopia, Koka	08°26N	39°02E	x		Eth 1
CBGP: M5659, M5678	Burkina-Faso, Niassan	13°07N	03°26W	x	x	BF 1,2
GenBank: HM635824	Cameroon, Gamnaga	10°57N	14°03E	x		Cam 1
GenBank: HM635825	Cameroon, Kongola	10°37N	14°25E	x		Cam 5
GenBank: HM635826	Cameroon, Kongola	10°37N	14°25E	x	x	Cam 4
GenBank: HM635827; CBGP: C344, C345, C352, C353	Cameroon, Maga	10°50N	14°57E	x		Cam 2, 6–9
CBGP: C358, C374; GenBank: HM635835, HM635836	Cameroon, Maga	10°50N	14°57E	x		Cam 10–13
GenBank: HM635828	Cameroon, Maga	10°50N	14°57E	x	x	Cam 3
MNHN: VV1998-060	Chad, Farcha	12°06N	15°03E	x	x	Cha 5
MNHN: VV1998-061, VV1999-213	Chad, Farcha	12°06N	15°03E	x		Cha 7,8
MNHN: 2000-060	Chad, Goz Djerat	10°59N	19°55E	x	x	Cha 4
MNHN: 2000-061	Chad, Goz Djerat	10°59N	19°55E	x		Cha 6
CBGP: M4134	Chad, Zakouma NP	10°44N	19°40E	x	x	Cha 1
CBGP: M4159, N3085	Chad, Zakouma NP	10°44N	19°40E	x		Cha 2,3
MNHN: VV1995-073	Egypt, breeding colony	?	?	x	x	Egy 1
GenBank: AF004569	Egypt, breeding colony	?	?	x		Egy 2
CBGP: M5240	Mali, Abeibara	19°01N	01°45E	x	x	Mal 6
CBGP: KM1039	Mali, Ansongo	15°40N	00°30E		x	Mal 29
CBGP: M5647	Mali, Bintagoungou	16°44N	03°44W	x	x	Mal 9
CBGP: M4060	Mali, Boulou	15°11N	09°31W	x	x	Mal 1
CBGP: M4189	Mali, Dialo	14°28N	11°30W	x	x	Mal 3
CBGP: M5645	Mali, Dianké	15°45N	04°39W	x	x	Mal 25
CBGP: M4260	Mali, Dirimbé	15°01N	02°54W	x	x	Mal 15
MNHN: VV1999-054	Mali, Edjerir	18°12N	01°24E	x		Mal 13
MNHN: VV1999-046	Mali, Edjerir	18°12N	01°24E	x	x	Mal 10
CBGP: M5385	Mali, Emnalhere	14°28N	04°05W	x	x	Mal 23
CBGP: M4605	Mali, Farabougou	14°53N	06°08W	x	x	Mal 17
CBGP: M4675	Mali, Farabougou	14°53N	06°08W	x		Mal 19
CBGP: M4680	Mali, Gono	15°03N	02°47W	x	x	Mal 20
CBGP: M4771	Mali, Gono	15°03N	02°47W	x		Mal 21
CBGP: M5649	Mali, Goubolabo	15°54N	03°59W	x	x	Mal 26
CBGP: M4065	Mali, Makana	15°08N	09°26W	x	x	Mal 2
MNHN: VV1999-076	Mali, Ménaka	15°55N	02°25E	x	x	Mal 14
CBGP: M5367	Mali, Niono	14°17N	05°59W	x	x	Mal 22
CBGP: KM1081	Mali, Ouatagouna	15°12N	00°42E	x	x	Mal 28
CBGP: M5639	Mali, San	13°22N	04°56W	x	x	Mal 24
CBGP: M4209	Mali, Sare Mama	14°53N	04°02W	x	x	Mal 4
CBGP: M4934	Mali, Sare Mama	14°53N	04°02W	x		Mal 5
CBGP: M5682	Mali, Tanda	15°46N	04°38W	x	x	Mal 27
MNHN: VV1999-049, VV1999-070	Mali, Tararabat	19°24N	01°14E	x		Mal 11,12
CBGP: M5265	Mali, Tidermène	17°01N	02°07E	x	x	Mal 7
CBGP: M5287	Mali, Tidermène	17°01N	02°07E	x		Mal 8
CBGP: M4589	Mali, Tirna	15°41N	04°44W	x	x	Mal 16
CBGP: M4619	Mali, Tirna	15°41N	04°44W	x		Mal 18
MNHN: VV1995-017	Mauritania, Chott Boul	16°37N	16°25W	x	x	Mau 1
MNHN: VV1995-041	Mauritania, Chott Boul	16°37N	16°25W	x		Mau 2
CBGP: N3214	Niger, Agadez	16°58N	07°59E	x		Nig 4
CBGP: N3222	Niger, Agadez	16°58N	07°59E	x	x	Nig 5
CBGP: N3143	Niger, Bosso	13°41N	13°18E	x	x	Nig 21
CBGP: N4251	Niger, Boumba	12°25N	02°50E	x	x	Nig 31
CBGP: N4211	Niger, Boumba	12°25N	02°50E	x		Nig 32
CBGP: N3266	Niger, Chétimari	13°10N	12°28E	x	x	Nig 10
CBGP: N4078, N4092	Niger, Chétimari	13°10N	12°28E	x		Nig 12,13
CBGP: N4094, N4152	Niger, Chétimari	13°10N	12°28E	x		Nig 24,25
CBGP: N4096	Niger, Djirataoua	13°24N	07°08E	x	x	Nig 16
CBGP: N4134	Niger, Djirataoua	13°24N	07°08E	x		Nig 17
CBGP: N4146	Niger, Gaya	11°53N	03°27E	x	x	Nig 19
CBGP: N4147	Niger, Gaya	11°53N	03°27E	x		Nig 20
CBGP: N3267	Niger, Gouré	14°03N	10°13E	x	x	Nig 7
CBGP: N4135	Niger, Guidimouni	19°24N	09°30E	x	x	Nig 18
CBGP: N3106, N3107	Niger, Guileyni	13°26N	02°42E	x	x	Nig 1,2
CBGP: N4271	Niger, Karey Kopto	12°33N	02°38E	x	x	Nig 33
CBGP: N4273	Niger, Karey Kopto	12°33N	02°38E	x		Nig 34
CBGP: N4093	Niger, Kojimairi	13°24N	11°05E	x	x	Nig 14
CBGP: N4098, N4109, N4133, N4154, N4159	Niger, Kojimairi	13°24N	11°05E	x		Nig 15, 27–30
GenBank: AF004571, AF004570	Niger, Kollo	13°21N	02°17E	x		Nig 23,24
CBGP: N3124	Niger, N’Guigmi	14°15N	13°06E	x	x	Nig 3
CBGP: N3144	Niger, Niamey	13°31N	02°05E	x	x	Nig 22
CBGP: N4001	Niger, Tabelot	17°36N	08°56E	x	x	Nig 9
CBGP: N3268	Niger, Tanout	14°57N	08°53E	x	x	Nig 8
CBGP: M5295	Niger, Tiloa	15°09N	02°04E	x	x	Nig 11
CBGP: N3219	Niger, Tondibia	13°34N	02°01E	x	x	Nig 6
CBGP: KB2807, KB2816	Senegal, Darou-Wolof	14°00N	14°47W	x		Sen 23,24
CBGP: JMD441, JMD447	Senegal, Gouniang	14°50N	12°26W	x		Sen 1,2
CBGP: KB932, KB933	Senegal, Kaolack	14°10N	16°07W	x		Sen 17,18
CBGP: KB1243	Senegal, Lampsar Peuhl	16°05N	16°20W	x	x	Sen 5
CBGP: KB1244	Senegal, Lampsar Peuhl	16°05N	16°20W	x		Sen 9
CBGP: KB924, KB925	Senegal, Lindiane	14°10N	16°09W	x		Sen 15,16
CBGP: KB3125, KB3126	Senegal, Mibess	15°25N	16°42W	x		Sen 25,28
CBGP: KB2407	Senegal, Ndya	14°34N	12°45W	x	x	Sen 11
CBGP: KB2408	Senegal, Ndya	14°34N	12°45W	x		Sen 12
CBGP: KB1191	Senegal, Niaga	14°49N	17°16W	x	x	Sen 10
CBGP: KB3143	Senegal, Pekh Tall	15°27N	16°24W	x		Sen 26
CBGP: KB3150	Senegal, Pekh Tall	15°27N	16°24W	x	x	Sen 27
GenBank: AF004572	Senegal, Richard Toll	16°28N	15°45W	x		Sen 29
CBGP: KB2765, KB2766	Senegal, Saré-Gayo	13°51N	13°58W	x	x	Sen 21,22
CBGP: KB1437	Senegal, Savoigne	16°09N	16°18W	x	x	Sen 6
CBGP: JMD645, JMD646	Senegal, Sinthiane Doudé	15°25N	12°57W	x		Sen 3,4
CBGP: KB2755	Senegal, Sinthiou-Maleme	13°49N	13°55W	x	x	Sen 19
CBGP: KB2758	Senegal, Sinthiou-Maleme	13°49N	13°55W	x		Sen 20
CBGP: KB2441	Senegal, Sinthiou-Doudé	14°11N	12°45W	x	x	Sen 13
CBGP: KB2442	Senegal, Sinthiou-Doudé	14°11N	12°45W	x		Sen 14
CBGP: KB1335, KB1336	Senegal, Wouro-Aïb	16°28N	15°37W	x		Sen 7,8
GenBank: EF128062, 128063, 128064	Sudan, Dongola	19°00N	30°29E	x		Sud 5–7
GenBank: EF128067, EF128068, EF128069	Sudan, El Sabagola	17°34N	33°26E	x		Sud 3, 10–11
GenBank: EF128070, EF128071, EF128072; DM: 9106	Sudan, El Sabagola	17°34N	33°26E	x		Sud 12–15
DM: 8993	Sudan, El Sabagola	17°34N	33°26E	x	x	Sud 4
DM: 9103, 9108	Sudan, El Suki	13°19N	33°54E	x	x	Sud 1,2
GenBank: EF128083, EF128084	Sudan, El Suki	13°19N	33°54E	x		Sud 26,27
GenBank: EF128073, EF128074, EF128075	Sudan, Khartoum	15°40N	32°35E	x		Sud 16–18
GenBank: EF128076, EF128077	Sudan, Khartoum	15°40N	32°35E	x		Sud 19,20
GenBank: EF128078, EF128079, EF128080	Sudan, Medani	14°23N	33°29E	x		Sud 21–23
GenBank: EF128081, EF128082	Sudan, Medani	14°23N	33°29E	x		Sud 24,25
GenBank: EF128065, EF128066	Sudan, Shandi	16°42N	33°29E	x		Sud 8,9
*Arvicanthis* cf. *niloticus* **						
MNHN: VV1996-009, VV1996-010	Kenya, Masai Mara NP	01°49S	35°20E	x	x	1,2
*Arvicanthis abyssinicus*						
GenBank: AF004567.1***	Ethiopia, Menagesha	09°03N	38°34E	x		1
GenBank: AF004566.1***	Ethiopia, Sululta	09°11N	38°46E	x		2
*Arvicanthis neumanni*						
GenBank: AF004574****	Tanzania, Berega	06°11S	37°09E	x		1
GenBank: AF004573****	Tanzania, Berega	06°11S	37°09E	x		2
*Arvicanthis ansorgei*						
CBGP: Leish NEG 024MS1	Burkina-Faso, Pissy	12°20N	01°35W	x	x	3
CBGP: M6068	Burkina-Faso, Toumbani	11°00N	00°58E	x	x	2
CBGP: M5619	Mali, Séniéna	10°50N	05°40W	x	x	1
*Arvicanthis* sp. (ANI-2)						
GenBank: HM635839	Cameroon, Gamnaga	10°57N	14°03E	x	x	ANI-2 1
GenBank: AF004584	CAR, Koumbala	09°14N	20°42E	x		ANI-2 2
*Arvicanthis* cf. *rufinus*						
GenBank: AF004582	Benin, Lokossa	06°38N	01°43E	x		1
GenBank: AF004583	Benin, Tanougou	10°49N	01°26E	x		2
non-*Arvicanthis* species						
*Aethomys chrysophilus* (GenBank: AJ604526.1)	Tanzania			x		
*Desmomys harringtoni* (GenBank: AF141206)	Ethiopia			x		
*Golunda ellioti* (GenBank: AM408338.1)	India			x		
*Lemniscomys striatus* (MNHN: ZM-2008-020)	Benin			x		
*Mastomys erythroleucus* (CBGP: M4136)	Chad				x	
*Mylomys dybowskii* (GenBank: AF141212.1)	Ivory Coast			x		
*Pellomys fallax* (GenBank: DQ022382.1)	Tanzania			x		
*Otomys irroratus* (GenBank: EU874434)	South Africa			x		
*Otomys sungae* (GenBank: JF795993)	?			x		
*Otomys tropicalis* (GenBank: JF795995)	?			x		
*Rhabdomys pumilio* (GenBank: AF533116)	South Africa			x		

**Figure 1:**
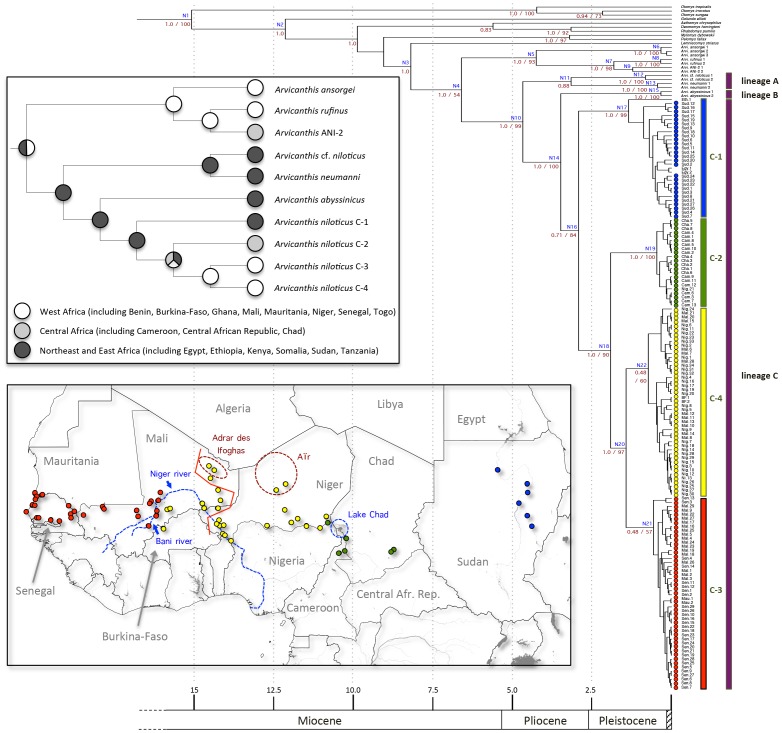
Time-calibrated tree resulting from the partitioned Bayesian analysis of the combined dataset. Age estimates correspond to the results of the BRC analysis using the fossil constraint with an exponential distribution. Labels on nodes correspond to the nodes listed in Table 2. Posterior probabilities (left) and bootstrap values (right) are also indicated for major nodes (values below<0.50 are not figured). On the bottom left (lower panel), a map is included to figure the localities of almost all sampled *Arvicanthis niloticus* specimens (with the exception of the specimen from Ethiopia and the two specimens from Egypt). Additional information on mountain and hydrogeographic formations of interest is also provided. A red line is also used to figure the distribution limits of the two distinct *A. niloticus* cytotypes (ANI-1a and ANI-1b; see text for details). On the right side, the general origin of *Arvicanthis niloticus* specimens is represented using circles filled with colours corresponding to those used on the map. In addition, vertical sidebars highlight the three major lineages (A, B and C) and the four major clades within the lineage C (C-1, C-2, C-3 and C-4). On the bottom left again (upper panel), the simplified phylogenetic topology of *Arvicanthis* studied here is represented with corresponding geographic origin of extant lineages as well as of MRCA as inferred by the ancestral area optimization under Mesquite (see text for details).

**Figure 2:**
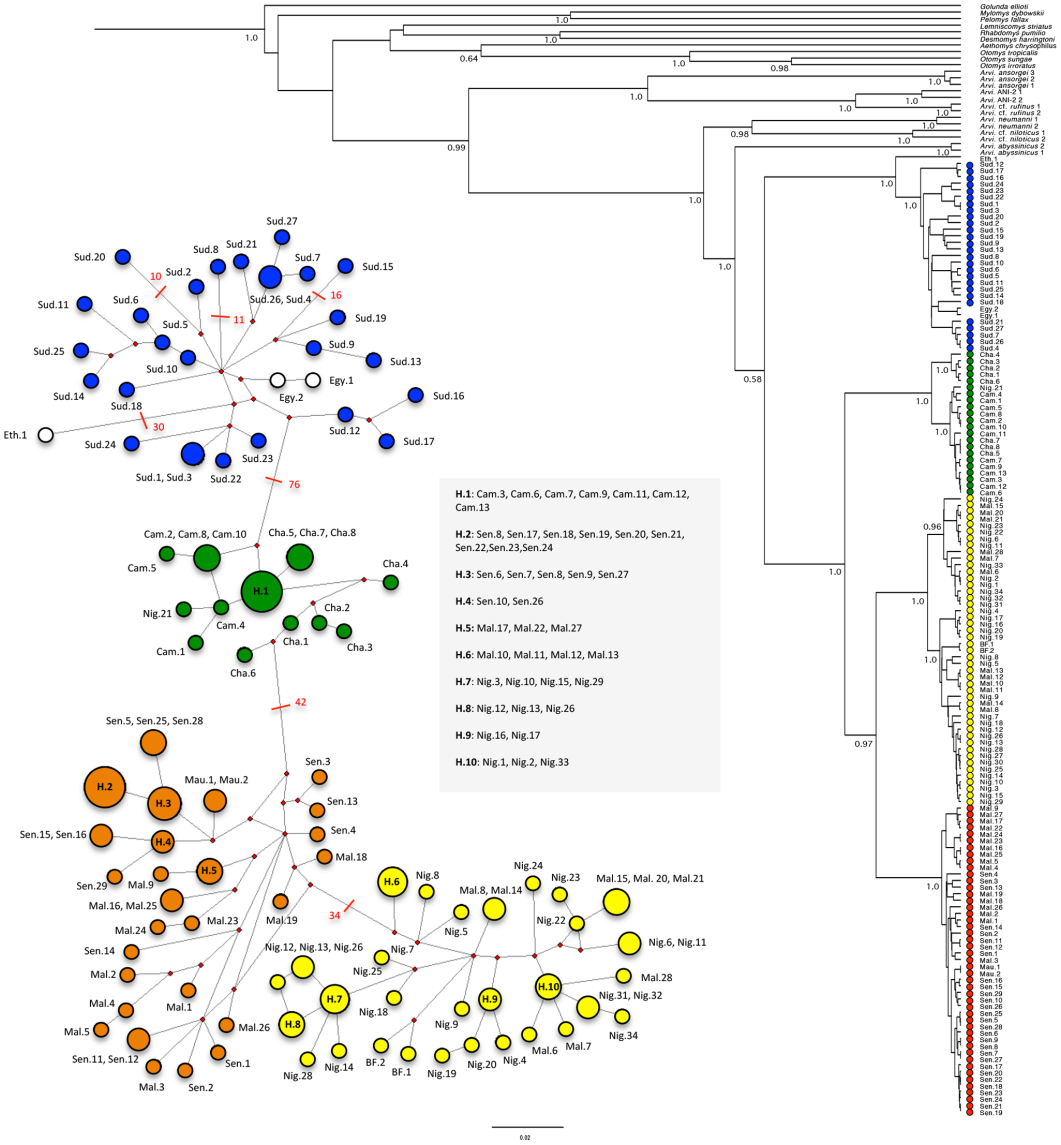
Haplotype network and phylogenetic tree resulting from the analysis of the cytb dataset. The haplotype network reconstruction takes into account missing data and gap so the inferred number of haplotype I higher than the one presented in Table 4. Red values on nodes indicate the inferred number of mutation steps between haplotypes or ancestral haplotypes (symbolized by a red node). The absence of value means that the number of steps is inferior to 10. The phylogenetic tree corresponds to the results of a Bayesian inference analysis (see text for details); posterior probabilities (PP) are indicated for major nodes (values below<0.50 are not figured). On the right side, the general origin of *Arvicanthis niloticus* specimens is figured using circles filled with different colours that directly refer to the map presented in Figure 1.

**Figure 3:**
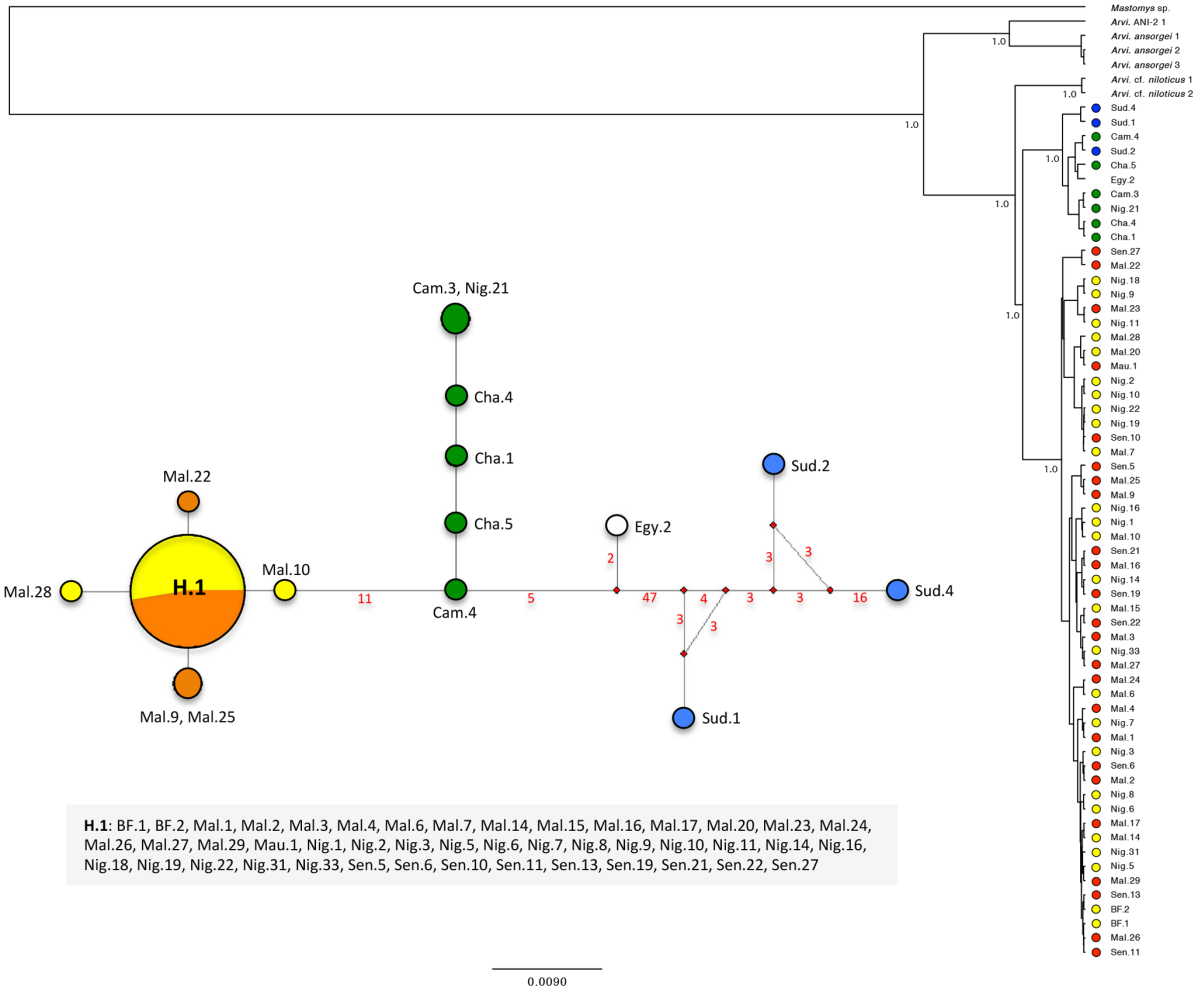
Haplotype network and phylogenetic tree resulting from the analysis of the Fib7 dataset. The haplotype network reconstruction takes into account missing data and gaps, so that the inferred number of haplotypes is higher than the one presented in Table 4. Red values on nodes indicate the inferred number of mutation steps between haplotypes or ancestral haplotypes (symbolized by a red node). The absence of value means that the number of step is equal to 1. The phylogenetic tree corresponds to the results of a Bayesian inference analysis (see text for details); posterior probabilities (PP) are indicated for major nodes (values below<0.50 are not figured). On the right side, the general origin of *Arvicanthis niloticus* specimens is figured using circles filled with different colours that directly refer to the map presented in Figure 1.

“CBGP”, “DM” and “MNHN” stand for Centre de Biologie pour la Gestion des Populations (Montpellier, France), Durban Natural Science Museum (Durban, South Africa) and Museum National d’Histoire Naturelle (Paris, France), respectively. Individuals’ labels corresponding to Figures 1 to 3 are also provided.

* referred to as *A. dembeensis* in [29];

** most probably referable to *A. somalicus* (cf. text for details);

*** referred to as *A.* cf. *abyssinicus* in [29];

**** referred to as *A.* cf. *somalicus* in [29].

### Ethics Statement

Each trapping campaign was validated by national and local authorities. At the French level, all sampling procedures were conducted by biologists from the IRD holding a certificate to carry out experiments on live animals (‘*certificat d’autorisation à expérimenter sur animaux vivants*’; agreement number C34-106, valid until December, 16^th^ 2016). The CBGP joint research unit is also holding an agreement to conduct experiments on live animals (‘*établissement agréé pour expérimenter sur animaux vivants’*; agreement number C34-169-1, valid until July, 25^th^ 2017). Within each country where sampling was performed, research was systematically made possible thanks to extant conventions between the IRD and local governments (see the regional sections as well as IRD Ethical Guidelines on the IRD website: www.ird.fr). Additional authorizations were not required because *Arvicanthis* species are considered as pest species (especially *A. niloticus*) and have no protected status (see IUCN and CITES lists). At the local level, traps were systematically set only after the agreement of the village head and the field owner was explicitly obtained. Moreover, in cultivated fields, traps were always posed on the edge of the exploited area, so that no damage could be cause to crops. Nile rats were caught alive in wire-mesh and Sherman traps. All animals were euthanized by cervical dislocation. Animals were treated in a humane manner, and in accordance with guidelines of the American Society of Mammalogists [43]. No ethic country-specific agreement could be obtained since countries where sampling occurred and the IRD have no ethics committee that oversees animal experimentation.

### DNA Extraction and Sequencing

Total genomic DNA was extracted using the Puregene DNA Purification Kit (Gentra Systems). The complete cytb mitochondrial gene was then amplified using primers H15915 and L14123 following procedures detailed in Lecompte *et al.*, [44]. The resulting PCR products were purified and then sequenced in both directions on a MegaBACE 1000 (Amersham Biosciences). Sequences were carefully checked by eye, and aligned using the ClustalW Multiple Alignment option implemented in BioEdit v.7.0.4.1 [45]. In total, our cytb dataset includes 169 sequences encompassing 146 *A. niloticus*, two *A.* cf. *niloticus*, 11 other *Arvicanthis* spp. and 10 representatives of other rodent genera (Table 1). The complete non translated intron 7 of the Fibrinogen nuclear gene was amplified using primers BFIBR1 and BFIBR2, following Seddon *et al.* [46]. These PCR products were purified and then sequenced in both directions by Macrogen (Seoul, Korea). In total, our Fib7 dataset groups 67 sequences that represent 60 *A. niloticus*, two *A.* cf. *niloticus* from Kenya, four representatives of other *Arvicanthis* species as well as one representative of genus *Mastomys*, to be used as an outgroup (Table 1). As stated previously, all individuals sequenced for Fib7 were sequenced for cytb (except for the *A. niloticus* specimen labelled ‘Mal 29′, and the representative of genus *Mastomys*), thus allowing us to build a combined dataset of 1,871 nucleotides (nt) encompassing 171 individuals. Importantly, all 10 countries that are represented for *A. niloticus* or *A.* cf. *niloticus* individuals in our cytb dataset are also represented in our Fib7 dataset. Similarly, 57 out of the 72 localities sampled in the *A. niloticus* or *A.* cf. *niloticus* cytb dataset were also sampled for Fib7 (see Table 1), thus providing a very similar and fully overlapping geographic coverage for both genes. All the sequences generated in this study were deposited in GenBank (accession numbers KF478244 to KF478310 for the Fib7 and KF478311 to KF478426 for the cytb).

### Phylogenetic and Dating Analyses

Bayesian inference (BI) was used to co-estimate phylogenetic relationships and divergence times using the BEAST v1.7.5 package [47]. BEAST uses Bayesian Markov Chain Monte Carlo (MCMC) procedures to approximate phylogenies and simultaneously infer nodes ages. To infer the time-calibrated phylogeny, we used the Bayesian relaxed clock (BRC) approach [48] implemented in BEAST. This method accounts for rate variation across lineages and assumes that substitution rates are uncorrelated across the tree (there is thus no *a priori* correlation between a lineage rate and that of its ancestor). To maximize the amount of available information, analyses were performed on the combined dataset, using specific sets of parameters for each gene [49]. Best-fit models of evolution for each gene were selected with jModelTest [50] using the Bayesian information criterion (BIC). For the molecular dating analyses, each gene was associated with a specific uncorrelated lognormal relaxed clock (ULRC) model. A coalescent model tree prior with a constant population size was also preferentially used to account for the fact that our trees mostly describe intra-specific relationships [51]. BEAST. xml files were also modified to implement the path-sampling (PS) procedure [52], which allows a better approximation of the marginal likelihood of runs [53]. In a complementary way, we carried out maximum likelihood (ML) analyses with the software RAxML v.7.0.8 [54] using default settings and 100 random-addition replicates. For the corresponding partitioned analyses, the same best-fit models of evolution (as in BI) were used. Clade support for ML was assessed with non-parametric bootstrap values (BV; 1,000 replicates were used).

Three distinct sets of calibration were used in this study. The first two sets rely on the oldest known fossil occurrence for the genus *Arvicanthis* [55]. This fossil (individual BPRP#76) is a relatively complete specimen found in Kenya, in the Lukeino formation, which was radiometrically dated at 5.9-5.7 million years ago (Ma; [55]). We used two distinct parametric distributions (exponential and lognormal) to set a minimum age of 5.7 Ma for the genus *Arvicanthis* (see [56], for more rationale on these settings). The corresponding 5.7 Ma minimum age constraint was enforced as follows: exponentialPrior mean = 5.0, offset = 5.444 (for the exponential distribution), and lognormalPrior mean = 1.0, stdev = 1.0, offset = 5.175 (for the lognormal distribution). For the third set of constraints we used the time-calibrated phylogeny of Lecompte *et al.* [42] to set a series of secondary calibrations for the nodes that are shared between our respective datasets. Though the use of secondary calibration is not advocated [56], [57], we conducted these analyses for comparison purpose. The fact that our outgroup choice was based on the result of the study of Lecompte *et al.* [42] allowed us to identify six shared nodes that were used for secondary calibrations. To do so, we used normal parametric distributions to set minimum and maximum ages for the corresponding nodes. These constraints were enforced as follows: (*i*) most recent common ancestor (MRCA) of *Arvicanthis* and *Aethomys*, normalPrior mean = 6.9 stdev = 1.0; (ii) MRCA of *Arvicanthis* and *Desmomys*, normalPrior mean = 8.4 stdev = 1.0; (iii) MRCA of *Arvicanthis* and *Lemniscomys*, normalPrior mean = 4.8 stdev = 1.0; (iv) MRCA of *Arvicanthis* and *Otomys*, normalPrior mean = 11.0 stdev = 1.0; (v) MRCA of *Desmomys* and *Rhabdomys*, normalPrior mean = 5.1 stdev = 1.0; (vi) MRCA of *Mylomys* and *Pelomys*, normalPrior mean = 3.85 stdev = 1.0.

For each calibration set (‘exponential distribution’; ‘lognormal distribution’; ‘secondary calibrations’), two distinct runs were carried out, each one with 50 million generations, default priors and trees sampled every 5000th generation. After applying a conservative burn-in of 12.5 million generations for each run, convergence of runs was further assessed by examining the effective sample size (ESS) of parameters with Tracer available at www.tree.bio.ed.ac.uk/software/tracer/. The resulting log and tree files were then combined using LogCombiner. We then directly estimated both node support (posterior probabilities: PP) and node age (median age estimates and 95% higher posterior densities: 95% HPD) using TreeAnnotator. Only nodes with posterior probabilities (PP) ≥0.95 were considered strongly supported [58]. Bayes factor (B_F_; [59]) were then estimated using the log files of the three distinct calibration sets, using scripts detailed in Baele *et al.* [53]. The resulting time-calibrated trees (one per calibration set) were further modified under Mesquite v2.75 [60] by pruning the most basal outgroup taxa. For comparison purpose, additional runs (same settings for the MCMC) were also conducted for the cytb dataset (169 taxa; 1,113 nt) and the Fib7 dataset (67 taxa; 758 nt).

Additional phylogenetic-based analyses were also conducted to precise the geographic origin of the various *Arvicanthis niloticus* populations. To do so, we relied on the tree resulting from the analyses of the combined dataset. This tree was further pruned under Mesquite in order to include one specimen for each of the major lineages of *A. niloticus*, and one specimen for each other *Arvicanthis* species. Character optimizations were then performed to infer the ancestral areas of distribution. Three major areas were categorized for this analysis: (i) ‘West Africa’, which includes Benin, Burkina-Faso, Ghana, Mali, Niger, Senegal, Togo; (ii) ‘Central Africa’, which includes Cameroon, Central African Republic, Chad; and (iii) ‘North-East and East Africa’, which here includes Egypt, Ethiopia, Kenya, Somalia, Sudan, Tanzania. We then performed a single state parsimony optimization with Mesquite in order to identify the geographic origin of the MRCA of each *A. niloticus* phylogroups.

### Genetic Differentiation, Genetic Diversity and Network Analyses

Analyses at the intraspecific level were performed on datasets that encompass only *A. niloticus* individuals (146 individuals for the cytb dataset and 60 individuals for the Fib7 one). The genetic differentiation between specimens belonging to the major phylogroups inferred by the phylogenetic analyses of the combined dataset was assessed for each gene using DnaSP v.5.1.0 [61]. However, the fact that DnaSP does not take into account missing data was problematic for the Fib7 dataset since it led to the exclusion of 113 nucleotide positions. Therefore, we analyzed a subset of the original dataset from which two sequences with a lot of missing data were removed [62]. In order to assess the level of genetic differentiation among the four phylogroups, we used three distinct statistics (F_ST_, K_ST*_, and S_nn_). The F_ST_ (fixation index) is a statistic that compares the level of diversity of randomly chosen alleles in a given population with those found in the entire geographical sample; the K_ST*_ is a statistic that takes into account the number of nucleotide differences between different haplotypes but does not give much weighting to large numbers of differences [63]; the nearest-neighbour statistic (S_nn)_ measures how often the nearest neighbours within a matrix of sequences originate from the same population [64]. Because these three indices are known to be more or less sensitive to specific dataset features (such as low level of genetic diversity or low sample size), we used them in combination to ensure a more robust detection of genetic differentiation [65]. For each statistics, a permutation test of 1,000 replicates was performed to assess the significance of the subdivision parameters. DnaSP was then used to infer the following parameters of genetic diversity: number of segregating sites *(S),* number of haplotypes (*h*), haplotypic (*Hd*) and nucleotide (π) diversities. We also performed neutrality tests for each gene using Tajima’s *D* [66] and Fu’s *F* statistics [67]. For the latter, values close to zero are expected for historically stable populations, whereas negative values would be indicative of recent population expansion.

Finally, networks analyses were conducted on the reduced cytb and fib7 datasets using the reduced median joining method [68], which has the ability to deal with missing data as well as to infer ancestral haplotypes. This method also performs well against, or outperforms other network approaches [69]. It was implemented using the software Network v.4.6.1.1 (http://www.fluxus-engineering.com), with ε set to 0 in order to minimize alternative median networks.

## Results

### Phylogenetic Analyses

The BIC returned a general time reversible (GTR) model+Γ as best-fit model for each gene. For all analyses (combined dataset, or Fib7 and cytb gene alone), convergence was reached as indicated by the high ESS values (>200) recovered for all runs.

Analyses of the combined dataset yield a similar topology whatever the method used (BI or ML; Figure 1). Overall the corresponding trees are well supported, and most interspecific nodes are supported by PP≥0.95 under BI or bootstrap values ≥70% under ML (Figure 1). Within the genus *Arvicanthis*, two main plurispecific clades emerge: specimens of *A. ansorgei*, *A.* cf. *rufinus* and ANI-2 cytotype (*sensu* [30]) form a well-supported monophyletic group (PP of 1.0/BV of 93%) whose sister assemblage contains (PP of 1.0/BV of 99%) representatives identified as *A. neumanni*, *A. abyssinicus*, *A.* cf. *niloticus* (two specimens from Kenya) and *A. niloticus sensu stricto* (i.e. all *A. niloticus* but the two latter Kenyan specimens) here below referred to as *A. niloticus*. Within the former group, *A. ansorgei* forms a first robust monophyletic lineage (PP of 1.0/BV of 100%), while the second one contains *A.* cf. *rufinus* and ANI-2 individuals (PP of 1.0/BV of 98%). Within the second main *Arvicanthis* assemblage, three lineages were clearly retrieved: lineage A (PP of 0.88/BV ≤50%) is basal and made of two *A.* cf. *niloticus* and two *A. neumanni* specimens from Kenya and Tanzania, respectively; then, lineage B (PP of 1.0/BV of 100%) is represented by the two *A. abyssinicus* specimens from Ethiopia; lineage C (PP of 0.71/BV of 84%) groups all *A. niloticus* (from Ethiopia, Sudan, Egypt throughout all other westward countries) specimens (Figure 1).

Central to the present study, lineage C splits into four phylogroups (C-1 to C-4), which also correspond to remarkably well-defined and exclusive geographic areas: (i) phylogroup C-1 (PP of 1.0/BV of 99%) contains all *A. niloticus* from Ethiopia, Sudan and Egypt; (ii) phylogroup C-2 (PP of 1.0/BV of 100%) groups all *A. niloticus* from Chad and Northern Cameroon, as well as one specimen from the Western bank of Lake Chad (Bosso) in Niger; (iii) *A. niloticus* individuals from West Mali to Senegal and Mauritania all gather in phylogroup C-3 (PP of 0.48/BV of 57%); (iv) specimens from phylogroup C-4 (PP of 0.48/BV of 60%) are all Nile grass rats originating from Niger, Burkina-Faso, North and East of Mali. In other words, in addition to a perfect match between genetic and geographic structure, a clear differentiation from East (phylogroup C-1 as sister to all remaining group) to West (phylogroups C-3 and C-4 as the most derived ones) is suggested. The latter pattern is also supported by the result of the character optimization of ancestral areas (see Figure 1), which indicates that the MRCA of all *A. niloticus* phylogroups is of East African origin. On a side note, it is also worthy to notice that individuals from Adrar des Ifoghas massif (specimens 'Mali.10’, ‘Mali.11’, ‘Mali.12’ and ‘Mali.13’) appear genetically very close to each other, while those from Aïr massif (specimens ‘Nig.4’, ‘Nig.5’ and *‘*Nig.9’) do not convincingly do so.

All the aforementioned lineages and phylogroups are recovered by the separate analysis of the cytb gene (see Figure 2), with moderate (PP of 0.58 for lineage C) to high supports (PP of 0.98 for lineage A, and PP of 1.0 for lineage B and phylogroups C-1, C-2, C-3 and C-4). By contrast, a less resolved and supported topology is inferred by the analysis of the Fib7 gene alone (see Figure 3), which only identifies two well-supported (PP of 1.0) clades within *A. niloticus*: the first one mixes together individuals that are found in previously mentioned phylogroups C-1 and C-2, while the second one gathers representatives from phylogroups C-3 and C-4. The two Kenyan representatives of *A.* cf. *niloticus* were found basal to all other *A. niloticus*.

### Divergence Time Estimates

Regarding dating analyses, the ‘secondary calibrations’ set yields younger age estimates (Table 2). The latter is problematic since the age of the oldest known *Arvicanthis* fossil (5.7–5.9 Ma) significantly predates the median age resulting from the ‘secondary calibrations’ calibration set (4.71 Ma). The two other calibration sets recover very similar median ages, which never differ from more than 1 Ma when considering Arvicanthini lineages. Because the ‘exponential distribution’ calibration set is significantly favoured by the corresponding B_F_ comparison (Ln B_F_ = −10881.60+11332.01 = 450.41), we chose to preferentially present the corresponding age estimates in Figure 1.

**Table 2:** Bayesian relaxed clock age estimates (Ma) based on three distinct calibration sets (fossil constraint with an ‘exponential distribution’, fossil constraint with a ‘lognormal distribution’, ‘secondary calibrations’).

Node	Ancestor of	Clade of interest	Fossil constr. (expon.)		Fossil constr. (lognormal)		Secondary calibrations	
			Median	95% HPD	Median	95% HPD	Median	95% HPD
1	*Otomys tropicalis* - *Arvi. niloticus* Sen.7		15.08	7.87–30.00	13.87	7.52–25.02	10.78	9.02–12.60
2	*Golunda ellioti* - *Arvi. niloticus* Sen.7	**Arvicanthini**	12.13	7.04–22.06	11.29	6.70–19.30	9.03	7.19–11.14
3	*Lemniscomys striatus - Arvi. niloticus* Sen.7		8.19	5.68–14.04	7.83	5.67–12.70	5.60	4.65–7.56
4	*Arvi. ansorgei* 1 *- Arvi. niloticus* Sen.7	***Arvicanthis*** ** spp.**	6.60	5.44 –11.41	6.52	5.27–10.24	4.71	3.40–6.07
5	*Arvi. ansorgei* 1 *- Arvi. rufinus* 1		4.25	1.67–7.85	4.16	1.89–7.00	3.00	1.75–4.36
6	*Arvi. ansorgei* 1 *- Arvi. ansorgei* 3		0.33	0.03–0.43	0.27	0.05–0.72	0.21	0.06–0.47
7	*Arvi. rufinus* 1 *- Arvi.* ANI2-1		1.81	0.65–3.71	1.67	0.65–3.36	1.25	0.62–2.06
8	*Arvi. rufinus* 1 *- Arvi. rufinus* 2		0.34	0.06–0.96	0.31	0.06–0.80	0.23	0.06–0.54
9	*Arvi.* ANI-2 1 *- Arvi.* ANI-2 2		1.22	0.40–2.66	1.07	0.36–2.33	0.83	0.31–1.51
10	*Arvi.* cf. *niloticus* 1 *- Arvi. niloticus* Sen.7		4.67	2.46–8.30	4.37	2.42–7.16	3.09	2.09–4.22
11	*Arvi.* cf. *niloticus* 1 *- Arvi. neumanni* 2	**lineage A**	3.16	1.20–5.89	2.83	1.15–5.11	2.03	1.04–3.20
12	*Arvi.* cf. *niloticus* 1 *- Arvi.* cf. *niloticus* 2		0.82	0.15–2.11	0.62	0.11–1.56	0.48	0.12–1.06
13	*Arvi. neumanni* 1 *- Arvi. neumanni* 2		0.44	0.07–1.05	0.40	0.06–1.13	0.31	0.06–0.73
14	*Arvi. abyssinicus* 1 *- Arvi. niloticus* Sen.7		3.48	1.67–6.46	3.20	1.59–5.46	2.33	1.49–3.34
15	*Arvi. abyssinicus* 1 *- Arvi. abyssinicus* 2	**lineage B**	0.33	0.05–1.05	0.30	0.05–0.89	0.22	0.04–0.59
16	*Arvi. niloticus* Eth.1 *- Arvi. niloticus* Sen.7	**lineage C**	2.92	1.42–5.42	2.72	1.59–5.46	1.97	1.25–2.82
17	*Arvi. niloticus* Eth.1 *- Arvi. niloticus* Sud.7	**phylogroup C-1**	1.35	0.50–2.72	1.25	0.56–2.41	0.92	0.50–1.50
18	*Arvi. niloticus* Cha.5 *- Arvi. niloticus* Sen.7		1.92	0.93–3.71	1.85	0.86–3.29	1.35	0.84–2.01
19	*Arvi. niloticus* Cha.5 *- Arvi. niloticus* Cam.13	**phylogroup C-2**	0.46	0.16–1.01	0.47	0.17–0.64	0.33	0.14–0.63
20	*Arvi. niloticus* Nig.30 *- Arvi. niloticus* Sen.7		1.43	0.66–2.76	1.16	0.54–2.12	0.88	0.55–1.38
21	*Arvi. niloticus* Sen.3 *- Arvi. niloticus* Sen.7	**phylogroup C-3**	0.57	0.35–1.49	0.49	0.20–0.97	0.34	0.19–0.57
22	*Arvi. niloticus* Nig.24 *- Arvi. niloticus* Nig.30	**phylogroup C-4**	0.73	0.22–1.23	0.63	0.31–1.19	0.45	0.27–0.71

The median age and 95% HPD of major nodes are reported for each calibration set. Clades of interest are highlighted using bold characters.

With respect to the age of Arvicanthini, a mid-Miocene origin was inferred with ages ranging from 11.29 Ma (95% HPD: 6.7–19.3; ‘lognormal distribution’ calibration) to 12.13 Ma (95% HPD: 7.04–10.924; ‘exponential distribution’ calibration). A Late Miocene origin was recovered for the genus *Arvicanthis* with ages ranging from 6.52 Ma (95% HPD: 5.27–10.24; ‘lognormal distribution’ calibration) to 6.6 Ma (95% HPD: 5.44–11.41; ‘exponential distribution’ calibration). The lineage encompassing all *A. niloticus* specimens (lineage C) appeared in the Late Pleistocene approximately 2.72–2.92 Ma. Several major splits then occurred in the Pleistocene (1.85–1.92 Ma, 1.16–1.43 Ma), leading to *A. niloticus* phylogroups C-1 to C-4.

### Genetic Differentiation, Genetic Diversity and Network Analyses

For the cytb gene, all statistics (F_ST_, K_ST*_, and S_nn_) recovered a significant level of genetic differentiation among the four major phylogroups (Table 3). By contrast, the level of genetic structure was lower for the Fib7 gene since no significant genetic structure was evidenced between C-1 and C-2, or between C-3 and C-4. For the latter, the statistics were not even computable because of the absence of genetic variation (when excluding gaps and positions with missing data) between the 50 sampled specimens of the two groups.

**Table 3:** Parameters describing genetic differentiation among phylogroups.

Gene	Groups	*F_ST_*	*K* * *_ST_*	*S_nn_*
Cytb	C-1 *vs* C-2	0.870***	0.344***	1.000***
	C-1 *vs* C-3	0.888***	0.389***	1.000***
	C-1 *vs* C-4	0.856***	0.303***	1.000***
	C-2 *vs* C-3	0.877***	0.400***	1.000***
	C-2 *vs* C-4	0.825***	0.309***	1.000***
	C-3 *vs* C-4	0.775***	0.314***	1.000***
Fib7	C-1 *vs* C-2	0.272 (ns)	0.247 (ns)	0.619 (ns)
	C-1 *vs* C-3	0.941**	1.000**	1.000**
	C-1 *vs* C-4	0.933**	1.000**	1.000**
	C-2 *vs* C-3	0.964***	0.915***	1.000***
	C-2 *vs* C-4	0.960***	0.924***	1.000***
	C-3 *vs* C-4	n/a	n/a	n/a

The following abbreviations were used:

* significant with *P*<0.05;

** significant with P<0.01;

*** significant with P<0.001. “n/a” indicates that it was impossible to compute the corresponding test statistic because of the lack of relevant data.

In contrast with the cytb gene (S = 182, h = 79, Hd = 0.985), genetic diversity was extremely low for the Fib7 gene (S = 9, h = 4, Hd = 0.253) (Table 4). For both genes, the highest levels of genetic diversity were found in the phylogroup C-1. Interestingly, this result was recovered despite the fact that the number of sampled individuals for phylogroup C-1 is lower than those of phylogroups C-3 and C-4 (and even C-2 when considering the Fib7 dataset). The results of the neutrality tests for the two genes showed that *A. niloticus* populations were stable over time. We only find evidence for expansion of populations’ size for the phylogroup C-1 (both Tajima’s *D* and Fu’s *F* statistics) and in the phylogroup C-3 (Fu’s *F* statistics).

**Table 4:** Parameters describing genetic diversity and genetic differentiation.

Gene	phylogroup (n)	*S*	*h*	*Hd*	π	*D*	*F*
Cytb	(All) (146)	182	79	0.985	0.0465	0.078 (ns)	−8.008 (ns)
	C-1 (30)	83	24	0.983	0.0151	−1.897*	−11.536*
	C-2 (22)	16	9	0.870	0.0055	0.318 (ns)	−0.695 (ns)
	C-3 (47)	32	24	0.946	0.0043	−1.713 (ns)	−14.579*
	C-4 (47)	57	22	0.946	0.0107	−0.863 (ns)	−2.781 (ns)
Fib7	(All) (58)	9	4	0.253	0.0026	−0.162 (ns)	3.101 (ns)
	C-1 (2)	1	2	1.000	0.0014	n/a	n/a
	C-2 (6)	1	2	0.600	0.0008	1.445 (ns)	0.795 (ns)
	C-3 (24)	0	1	0.000	0.0000	n/a	n/a
	C-4 (26)	0	1	0.000	0.0000	n/a	n/a

The following abbreviations were used: number of sequences per phylogroup (n); number of polymorphic sites (*S*); number of haplotypes (*h*); haplotypic diversity (*Hd*); nucleotide diversity (π); Tajima’s *D* statistic (*D*); Fu’s *F* statistic (*F*).

Network analyses recovered a relatively congruent pattern for both genes (Figure 2 and Figure 3), in which the haplotypes from East Africa (phylogroup C-1) are connected with haplotypes from Central Africa (phylogroup C-2), which are further connected with haplotypes from West Africa (phylogroups C-3 and C-4), thus indicating a clear East to West pattern of colonization. Furthermore, the cytb dataset suggests that the genetic pool that is now present in the eastern part of West Africa (phylogroup C-4) originated from the haplotypic population that currently lies in the western part of West Africa (phylogroup C-3).

## Discussion

All *Arvicanthis* individuals cluster in a monophyletic clade, whose sister group is *Lemniscomys striatus*, in agreement with Lecompte *et al*. [42]. Within *Arvicanthis*, three distinct clades are retrieved. A first one, that would be the sister group of all other *Arvicanthis* specimens studied here, comprises two subgroups of specimens: one includes individuals of *A. ansorgei* and the other contains individuals of *A.* cf. *rufinus* as well as those referred to as ANI-2. Such an association was already retrieved and discussed by Ducroz *et al*. [29], Volobouev *et al*. [70], and Dobigny *et al*. [34], and it will thus not be commented further here.

A second clade comprises two specimens of *A. neumanni* and two *A.* cf. *niloticus* specimens from Kenya. The two specimens labelled *A. neumanni* (from Tanzania) are those studied under the name *A. somalicus* by Ducroz *et al*. [29] who found them to be characterized by a 2N = 54/NFa = 62 karyotype. Ducroz [27] further proposed that they correspond to *A. neumanni*, as acknowledged by Castiglia *et al*. [32] to distinguish them from the true *A. somalicus* that they considered as having a karyotype with 2N = 62 and NFa = 62–63. As in Ducroz *et al*. [29], these two *A. neumanni* specimens have a sister-group relationship with the *A. niloticus*/*A. abyssinicus* clade. Yet, they here share this position with two *A.* cf. *niloticus* from Kenya, that were also karyotyped by Ducroz [27] as 2N = 62/NFa = 62. The clear distinctness of these two *A.* cf. *niloticus* from the remaining *A. niloticus* specimens on molecular grounds strongly suggests that another name should be given to them. The latter may well be *A. somalicus* since their karyotypes seem to match quite well the one proposed by Baskevich & Lavrenchenko [71] for this species (for further comments, see [32]).

The third main clade is characterized by the two individuals of *A. abyssinicus* that lie in a basal position relative to all other *Arvicanthis niloticus*. This result, also found using a much smaller sample of *A. niloticus* specimens by Ducroz *et al*. [29], tends to consolidate the sister status of *A. abyssinicus* and *A. niloticus*. The two species are sympatric, but apparently not syntopic, in Ethiopia where the former is considered an endemic of the High Plateaus, whereas the latter would range at lower altitudes, west of the Rift Valley [24]. The remaining part of this clade corresponds to *Arvicanthis niloticus*, whose phylogeography is discussed in detail below, thanks to a large and wide-ranging sample.

It was previously shown that *A. niloticus* may display two distinct karyomorphs differing by an inversion: ANI-1a with 2N = 62/NFa = 62 and ANI-1b with 2N = 62/NFa = 64 on the Eastern and Western parts of the species range, respectively, with no geographic overlap ( [30]; see Fig. 1). Nevertheless, the two chromosomal lineages were also found paraphyletic on DNA grounds, and the taxonomic significance of this chromosomal mutation has already been questioned [30]. Since then, reasonably large series of animals were karyotyped on the Eastern part of *A. niloticus*’ range (Sudan: 33; Northern Cameroon: 34) and, accordingly, they were found with the ANI-1a karyotype. Moreover, the geographic distributions of ANI-1a and ANI-1b (see Fig. 7 in [30]) resemble that of the genetic clades retrieved here, with C-3 range (this study) widely overlapping ANI-1b’s one [30]. However, this geographic match is imperfect since C-3 does not extend beyond the Bani River, while ANI-1b reaches the Adrar des Ifoghas massif (Northern Mali) and South-Western Niger (i.e., Anderamboukane). More precisely, several localities were included in both the cytogenetic and the molecular studies (e.g., Menaka, Ouatagouna and Tararabat, all in North-Eastern Mali) and provided animals with the ANI-1b karyotype but belonging to the C-4 (and not C-3) mtDNA clade. In addition, the two cytotypes were found sympatric in Sare Mama (Central Mali; Granjon, unpubl. data), and one heterozygous individual (2N = 62/NFa = 63) was trapped in Gaya (Southern Niger; Dobigny, unpubl. data). Taking into account the strict geographic exclusion of the C-3/C-4 mtDNA clades observed here, such an imperfect geographic match between chromosomal and molecular patterns may be due to asymmetric introgression between the nuclear (here the karyotype) and mitochondrial genomes following secondary contact of clades C-3 and C-4. Unfortunately, the resolution power offered by the Fib7 gene is too weak here to test for this hypothesis. Karyotypic vs. mtDNA profiles mismatch could also be due to incomplete lineage sorting of a long-standing inversion polymorphism, as already observed in another murid rodent genus, namely *Mastomys*, within the same regions [16]. If true, this would imply that this inversion polymorphism has been segregating in *A. niloticus* since at least the C-3/C-4 divergence, ∼1.16–1.43 Ma ago, hence is hemiplasic [72]. These two explanations (introgression and incomplete lineage sorting) are not mutually exclusive. Moreover, the detection of animals with heterozygous karyotypes (i.e., ANI-1a×ANI-1b) within the range of both C-3 and C-4 mtDNA clades suggest at least persisting nuclear gene flows between these two lineages.

The present work is the first one to include both mitochondrial and nuclear DNA sequences for phylogeographic purposes in African rodents. These two genes led to rather congruent patterns, although the nuclear dataset was much less phylogenetically informative. This is expected since nuclear DNA in coding regions usually evolves under slower mutation rates. Nevertheless, the use of Fib7 appeared informative here, and insures the absence of no sex-associated bias (such as male-biased dispersal) in our study.

The first striking result that was obtained here is that the TMRCA for all *A. niloticus* lineages (2.72–2.92 Ma) is markedly older than those of all nine other African rodent species that were investigated so far (all between 1.25 Ma in *Acomys chudeaui* and 0.31 Ma in *Praomys tullbergi*; see references above). This may be attributable to possible biases in previous dating analyses, which likely result from the overuse of questionable secondary calibrations such as the classical *Mus/Rattus* split at 12 Ma [73], in association with the use of methods that do not always account for rate variation across lineages. It could also be due to the existence of non-sampled lineages in the other species (for instance due to either true sampling bias or basal lineages’ extinction), or to a higher mutation rate in the *Arvicanthis* mitochondrial genome. Yet, geographic coverage appears really comparable in most investigations, with several of the previous studies also considering numerous localities all along from Western to Eastern Africa (e.g., [13], [15]). In addition, mtDNA-based analyses of higher-systematic levels within murids never detected any atypical molecular features or particularly long branches in *Arvicanthis* (e.g., [41]–[42], [44], [74]). As a consequence, and waiting for further proper comparisons between species, we suggest that *A. niloticus* can reasonably be considered as a truly rather ‘old’ species, already following its own trajectory with a first major split ∼2 Ma ago. Our results are significantly older than those inferred by Abdel Rahman and colleagues [33]. While taxonomic and geographic coverage as well as calibration and inference methods are quite different in both studies, thus making a proper comparison difficult, such a discrepancy is probably due to their use of a younger *Arvicanthis* fossil supposed to be 5-4 Myr old [33], whereas we relied on a 5.9-5.7 Myr old one [53]. Interestingly, our dates fit perfectly well with one of the major faunal turnover that was associated with grasslands expansion and increased adaptation to open habitats (2-1.8 Ma; [75]).

Furthermore, there is little doubt from our results that *A. niloticus* centre of origin is Eastern Africa. Indeed, the lineages that are the most closely related to unambiguously identified *A. niloticus* phylogroups (i.e. C-1 to C-4) all originate from this region (Table 1 and Fig. 1): *A. abyssinicus* (Ethiopia), *A. somalicus* (represented here by the two ‘*A.* cf. *niloticus*’ specimens from Kenya; see above) and *A. neumanni* (Tanzania). The corresponding character optimization of ancestral areas also supports this hypothesis (Fig. 1). At some point, it is also the case of the results of the genetic diversity analyses, which recovers a higher level of genetic diversity for the phylogroup C-1. Thanks to the results of the network analyses, we can also infer a more precise pattern for *A. niloticus* phylogroups. Overall, there is a clear East to West differentiation of populations, with Egyptian and Sudanese individuals (phylogroup C-1) being connected to Chadian or Northern Cameroonese (phylogroup C-2) populations. Unfortunately, a wide sampling gap exists between Sudanese (phylogroup C-1) and Chadian (phylogroups C-2) samples which precludes any precise localization of the contact zone between these two lineages. Surprisingly, the clade from Central Africa (phylogroup C-2) is genetically more related to a lineage that is not in direct contact (phylogroup C-3), thus suggesting that the ancestors of these lineages were likely isolated during a past episode. After this episode, the eastern part of West Africa was recolonized by another population (phylogroup C-4), which is in contact with phylogroup C-3 in Central Mali (more precisely along the Eastern side of the Niger River valley) and in contact with phylogroup C-2 in the Lake Chad surroundings.

As already suggested for many other rodent as well as non-rodent African organisms (see below, and Table S1), such a pattern, which is referable to phylogeographic category I of Avise [76], strongly suggests that extant genetic structure in *A. niloticus* results from the divergence of allopatric populations, either through ecological local adaptation, through vicariance (i.e. on each side of persisting geographic barriers) or through isolation in refuges and subsequent dispersal until secondary contact. West African steppes and savannas represent a rather homogenous habitat from Senegal to the Red Sea. This is why the East-West differentiation of *A. niloticus* lineages can hardly being accounted for by ecology-driven local adaptive processes. Furthermore, the numerous individuals that were trapped in very close localities but that yet belong to different genetic clades (e.g., in Central Mali and around Lake Chad) poorly support the local adaptation hypothesis. In the same manner, although putative geographic barriers that currently delimit *A. niloticus* intraspecific clades (Niger River, Lake Chad basin) were identified, vicariance appears as an inaccurate explanation here since no admixture at all was detected in our dataset. Indeed, this would imply that propagules crossed each of those barriers only ‘once’ and then remained reproductively isolated for hundreds thousand years, something that sounds highly improbable.

On the contrary, the refuge theory [77] fits well to the phylogeographic pattern observed in the Nile grass rats: lineages’ splits all occurred during the Pleistocene which is characterised by deep climatic variations that induced extensive modifications of the open grasslands habitat range in Sub-Saharan Africa. Indeed, some 3 million years ago, the African climate started to get generally cooler and drier, along with successive arid-pluvial cycles, more or less following glacial-interglacial ones at higher latitudes [78]–[81]. Whatever sudden [78], [79] or progressive [80], these recurrent aridification events have led to cyclic contractions of the moistest habitats (i.e., riverine and tropical forests, swampy areas), which were reduced to refugial patches in several instances during the Plio-Pleistocene surrounded by savannah-forest mosaic landscapes [82]. In parallel, arid phases induced wide-scale expansion of more xeric and open habitats. These climatic oscillations translated into deep modifications of all major biomes distribution ([79]–[81], [83]–[85]; among many others). In particular, C4 grasses, which are considered as valuable biomarkers of open grassland habitats, emerged during the Upper Miocene but became a major component of African biomes only from the Plio-Pleistocene [86]. Recurrent phases of grassland expansion following Pleistocene climatic cycles were accompanied by major turnovers of mammalian faunas towards more numerous and more abundant open habitat-adapted species [85]. Conversely, phases of savannah fragmentation have triggered intra- and inter-specific diversification ( [10], and references below). These variations in grassland habitats of the Sahelian region during the Pleistocene have probably had major consequences on *A. niloticus* evolution since this species is highly specialized in this habitat type: its diet is mainly herbivorous, and it also forages along runways through matted grass around its nests which are mainly made of intermingled blades of grass themselves [25], [87].

The refuge theory is also strongly supported by an increasing number of studies dealing with other Sub-Saharan rodent species (see below as well as Table S1), and where geographic distributions perfectly or almost perfectly match with genetic assemblages. All these studies similarly point towards isolation and genetic differentiation within refuges during unfavourable periods, with subsequent dispersion phases during favourable ones. Secondary contacts then tend to stabilize around strict (or even partial) geographic barriers that stop (or slow down) dispersal and gene flow –the so-called suture zones *sensu* Hewitt [88]. Of course, the nature of refuges, favourable/unfavourable periods and/or barriers depend on species-specific characteristics.

For instance, the Sahelo-Saharan spiny mouse *Acomys chudeaui* was most probably restricted to rocky areas that were surrounded by sand deserts during arid episodes, but may have dispersed through steppe-like environments during less arid periods [18]. The humid habitat-adapted *Mastomys huberti* underwent isolation around water-body relics during arid phases, while it colonized hydrographic basins of Senegal, Mali and Guinea during humid ones [19]. As far as forest-dwelling rodent species are concerned, they are expected to be trapped in forest relic patches during arid episodes but to extend during forest expansions associated with humid periods (e.g., *Praomys tullbergi* and *Praomys rostratus*: [21]; *Praomys misonnei*: [22],[23]). Similarly, populations of open tree savannah species would diverge allopatrically during moist episodes, when fragmented throughout extended savannah-forest mosaic landscapes (e.g., *Praomys daltoni*: [14]). On the contrary, species from the drier scrub savannah and steppes are thought to be isolated when surrounded by forests during the moistest phases, and then to disperse widely with expanding open grasslands (*Mastomys erythroleucus*: [15]; *Lemniscomys striatus*: [13]). *A. niloticus* is to be considered as belonging to the latter category.

Geographic barriers to dispersal were also identified in most of African rodent phylogeographic investigations. Once again, many similarities appear between species-specific case studies. The tectonic complex of the Rift Valley was identified as a contact zone between parapatric phylogroups in *L. striatus* [13] and *Mastomys natalensis* [17]. Similarly, the relationships between some hydrographic features and genetic structure have been documented in several Sub-Saharan rodent species (e.g., *Taterillus* spp.: [89]; *M. erythroleucus*: [15], [16]; *L. striatus*: [13]; *P. tullbergi*: [21]; *P. misonnei*: [22], [23]; *P. daltoni*: [14]). In particular, it is striking to see how the same rivers and/or lakes have been pointed out as putative barriers in several taxa. In regards to the present study, this is notably the case for the Niger River valley that tends to separate clades C-3 and C-4 of *A. niloticus*, as well as several parapatric clades within (*L. striatus*: [13]; *M. erythroleucus*: [15], [16]; *P. daltoni*: [14]) and between (*Taterillus* spp.: [89]) other unrelated rodent species. In the same manner, Lake Chad and its surroundings coincide with the contact zone between *A. niloticus* clades C-2 and C-3, while this area also signs genetic hiatus in *Mastomys erythroleucus* [15], [16], *Gerbillus nigeriae* [90] and *Taterillus* spp. [89]. Besides, it has already been suggested that the latter region constitutes a ‘phylogeographic crossroad’, i.e. a centre of diversification where faunas may have diversified following the recurrent Plio-Pleistocene cycles of transgression/regression of the Palaeolake Chad [34], [91].

So, in essence, ancestral *A. niloticus* populations would have diverged allopatrically during humid periods within at least four different Pleistocene refuges of open habitat. They would have dispersed during more arid ones until secondary contacts around geographic barriers such as the Niger River and the Lake Chad basins. The precise timing of isolation within Pleistocene refuges and of dispersal outside these refuges is not feasible here since molecular inferences of dates for such events are associated with confidence intervals that are usually of the same magnitude -when not larger- than Pleistocene climatic cycles themselves, thus precluding any robust conclusion. Also, the putative existence of a third barrier eastwards will require further sampling in Eastern Chad and Sudan in order to identify the precise geographic range of clades C-1 and C-2.

Finally, although we did not attempt to investigate formally sub-structure within each clade, we clearly retrieve a well-supported group of individuals that all originate from Adrar des Ifoghas in Mali (4 specimens from 2 different localities). No such signal could be obtained for individuals from Aïr in Niger (3 specimens from 2 different localities), thus supporting a previous hypothesis [92], [93] that the Malian Adrar may represent a more isolated Sahelian refuge within the Sahara desert than the Aïr.

Interestingly, the picture drawn from studies on rodents often resembles those obtained on other mammals that we are aware of: refuges and allopatry during successive climatic cycles in the past were advocated to explain the differentiation of genetic lineages in cercopithecine monkeys (e.g., [94], [95]), baboons [96], hyenas [97], wild dog [98], common warthog [99], giraffe [100], buffalo [101] and many large antelopes like topi, hartebeest [102], [103], impala, kudu [104], waterbuck [105], kob [106], African bushbuck [107], common eland [108] and roan [109]. Similar conclusions were also reached in plants (e.g., shea tree: [110]; giant lobelia: [111]; coffee tree: [112]), insects (e.g., maize stalk borer: [113]), reptiles (e.g., puff adder: [114]; Southern rock agama: [115]) and birds (e.g., starred robin: [116]; ostrich: [117]). Zoogeographic barriers to dispersal have also been pointed out in several species, including the Rift Valley (e.g., lion: [118]; giraffe: [100]; wildebeest: [102]; waterbuck: [105]; bushbuck: [107]; maize stalk borer: [113]; giant lobelia: [111]) and great rivers (e.g., [95]).

As a whole, this reaches what was inferred for other continents, especially the well-studied Europe and North America [119]–[121]. Sub-Saharan Africa was once claimed to be understudied [119], but data have accumulated during the last decade (reviewed in [10], and references hereabove). Among studies that have concerned ungulates, a clear sampling bias towards Eastern and Southern Africa is obvious. West African megafauna’s phylogeography remains very poorly documented and this part of the continent is usually dramatically absent from most datasets: Lorenzen and colleagues’ recent review [10] clearly illustrates such a gap of knowledge, with several highly differentiated ungulate lineages west of the Rift Valley (see their Figure 3) that, unfortunately, are documented through extremely reduced number of localities (but see [101], for a slightly higher number of sampling sites in buffaloes). This emphasizes the importance of studies conducted on rodents that, in the contrary, have mainly focused on West and Central African deserts, grasslands and forests. In particular, the present study adds to the three other available ones that are typical of open grasslands in West and Central Africa, namely *Mastomys natalensis* [17], *M. erythroleucus* [15] and *Lemniscomys striatus* [13]. Together, these latter works allow us to draw an integrative picture of West and Central African open habitats history during the Pleistocene, something that could not be reached with ungulates models. In particular, one refuge was hypothesised in West Africa (i.e., between Senegal and the Rift Valley) on the basis of large mammals data [10], while at least three and potentially four major ones can be speculated from patterns obtained in these four rodent species: one most probably westward of the Niger River, one somewhere between the Niger River and the Lake Chad, and one or two between the Lake Chad and the Nile River. Nevertheless, the precise locations of these Pleistocene refuges for open habitat species are still to be precisely assessed.

## Supporting Information

Table S1 **Summary of previous phylogeographic studies conducted on Sub-Saharan African rodents.** The species, their preferred habitat, the molecular marker used, the sample size, the methods for date inference, the major evolutionary events as well as putative corresponding geographic aspects are indicated. References can be found in the main text.
(DOCX)
