# Correction: Mitochondrial and Nuclear Genes-Based Phylogeography of *Arvicanthis niloticus* (Murinae) and Sub-Saharan Open Habitats Pleistocene History

**DOI:** 10.1371/annotation/a34daea8-8922-4eb0-8b4e-b0f9dbfd28ca

**Published:** 2013-12-13

**Authors:** Gauthier Dobigny, Caroline Tatard, Philippe Gauthier, Khalilou Ba, Jean-Marc Duplantier, Laurent Granjon, Gael J. Kergoat

There is an error in the "Results" section under the subheading "Divergence Time Estimates." The first sentence of the second paragraph should read: With respect to the age of Arvicanthini, a mid-Miocene origin was inferred with ages ranging from 11.29 Ma (95% HPD: 6.7–19.3; ‘lognormal distribution’ calibration) to 12.13 Ma (95% HPD: 7.04-22.06; ‘exponential distribution’ calibration). 

