# Supplementary material for: Mitochondrial and Nuclear Genes-Based Phylogeography of Arvicanthis niloticus (Murinae) and Sub-Saharan Open Habitats Pleistocene History
Source: PLoS One. 2013 Nov 1;8(11):e77815. doi: 10.1371/journal.pone.0077815 (PMC3815218; doi:10.1371/journal.pone.0077815)
Supplement: Table S1 — Summary of previous phylogeographic studies conducted on Sub-Saharan African rodents. The species, their preferred habitat, the molecular marker used, the sample size, the methods for date inference, the major evolutionary events as well as putative corresponding geographic aspects are indicated. References can be found in the main text. (DOCX) [file pone.0077815.s001.docx]

**Supplementary Table S1**. Summary of previous phylogeographic studies conducted on Sub-Saharan African rodents. The species, their preferred habitat, the molecular marker used, the sample size, the methods for date inference, the major evolutionary events as well as putative corresponding geographic aspects are indicated. References can be found in the main text.

| Species (habitat)* | Marker** | Sample*** | Dating method | Events | Dates | Geographic aspects**** |
| --- | --- | --- | --- | --- | --- | --- |
|  |  |  |  |  |  |  |
| *Acomys johannis* (SSRS) | cytb (1093) Dloop (563) | 38 (90, 51) 37 (83, 47) | TMRCA in BEAST (cytb only) based on *Mus/Rattus* divergence | TMRCA clade I | 0.062 | SR: Fachi, Tenere desert, Niger |
|  |  |  |  | TMRCA clade II | 0.25 | SR: Central Niger, including Aïr Mountains |
|  |  |  |  | TMRCA clade III | 0.283 | SR: SW Niger, Mali (including Adrar des Ifoghas), SE mauritania |
|  |  |  |  | TMRCA clade IV | 0.121 | SR: Mauritanian Adrar |
|  |  |  |  | split clade II vs. III | 0.411 | CZ: Ighazer plains (Niger) down south to Nigeria |
|  |  |  |  | split clade IV vs. II+III | 0.704 | CZ: NE-SW axis throughout Mauritania |
| *Arvicanthis niloticus* (SOG) | cytb (1113) Fib7 (758) | 69 (146, 79) 54 (60, 4) | TMRCA in BEAST based on oldest *Arvicanthis* fossil as well as secondary calibrations | split clade 1 vs. 2+3+4 | 2.92-2.72 | CZ: somewhere between Lake Chad and Nile Valley |
|  |  |  |  | split clade 2 vs. 3+4 | 1.92-1.89 | CZ: Lake Chad surroundings |
|  |  |  |  | split clade 3 vs. 4 | 1.46-1.16 | CZ: Bani River, in the Niger River hydrographic basin |
| *Lemniscomys striatus* (SOG) | cytb (838) | 42 (128, 75) | Coalescence in RRTree and TMRCA in MDiv based on *Mus/Rattus* divergence | split within clade IV | 0.64-0.44 | CZ: within Ivory Coast |
|  |  |  |  | split within clade II | 0.54-0.47 | CZ: within Rift Valley |
|  |  |  |  | split within lineage I | 0.49-0.31 | CZ: within Democratic Republic of Congo |
|  |  |  |  | split lineages I vs. II | 0.90-0.68 | CZ: W Rift Valley |
|  |  |  |  | split lineage III vs. I+II | 0.99-0.58 | CZ: Niger River |
|  |  |  |  | split lineage IV vs. I+II+III | 1.19-0.73 | CZ: Volta River |
| *Mastomys erythroleucus* (SOG) | cytb (1115) | 59 (215, 135) | TMRCA in BEAST based on *Mus/Rattus* divergence | TMRCA clade A | 0.22 | SR: W Burkina-Faso westwards to Senegal |
|  |  |  |  | TMRCA clade B | 0.68 | SR: W Burkina-Faso weastwards to Bani River and Lake Chad |
|  |  |  |  | TMRCA clade C | 0.43 | SR: Chad and Uganda |
|  |  |  |  | TMRCA clade D | 0.18 | SR: Sudan and Ethiopia |
| *Mastomys huberti* (HZ) | cytb (1111) | 41 (141, 65) | Molecular clock based on secondary calibrations | split within clade I | 0.17 | Southern part of the Niger River Inner Delta |
|  |  |  |  | split clade I vs. II | 0.60 | Senegal + Gambia Rivers basins vs. Niger River basin |
|  |  |  |  | split clades I+II vs. III+IV | 0.93 | Senegal + Gambia Rivers basins vs. Niger River basin (imperfect) |
| *Mastomys natalensis* (SOG) | cytb (1115) | 121 (209, 158) | TRMCA in BEAST based on oldest *Praomys* and *Mastomys* fossils | TMRCA clade A-I | - | SR: ~West of the Niger River |
|  |  |  |  | TMRCCA clade A-II | - | SR: between W and E Rift Valley |
|  |  |  |  | TMRCA clade A-III | - | SR: somewhere between Rift Valley and central Niger |
|  |  |  |  | split clade A-II vs. A-III | 0.87 | CZ: Rift Valley |
|  |  |  |  | split clade B-V vs. B-VI | 0.85 | CZ: within Tanzania |
|  |  |  |  | split clade I vs. II+III | 1.02 | CZ: central Niger down South to Nigeria |
|  |  |  |  | split clade IV vs. V+VI | 1.01 | CZ: within Kenya and Tanzania |
|  |  |  |  | split clade A vs. B | 1.16 | CZ: Rift Valley |
| *Mus (Nannomys) minutoides* (SOG) | cytb (846) | 15 (66, 15) | TRMCA in BEAST based on secondary calibrations | TMRCA clade WCA | 0.14 | SR: W Central Africa |
|  |  |  |  | TMRCA clade GIC | 0,18 | SR: Guinea and Ivory Coast |
|  |  |  |  | TMRCA clade SAK | 0,79 | SR: Southa Africa to Kenya |
|  |  |  |  | TMRCA clades WAC+SAK | 0,96 | - |
| *Praomys* cf. *daltoni* (SOG) | cytb (987) microsats (7) | 91 (137, 104) | TMRCA in BEAST based on secondary calibrations | TMRCA clade A | 0.49 | SR: between Volta and Niger Rivers |
|  |  |  |  | TMRCA clade C1 | 0.39 | SR: N Cameroon |
|  |  |  |  | TMRCA clade C2 | 0.44 | SR: Ghana, Benin and Togo |
|  |  |  |  | split clade C1 vs. C2 | 1.04 | SR: ~Niger River |
|  |  |  |  | split clade B vs. C+C1+C2 | 3.16 | SR: somewhere between lower Niger River and N Cameroon |
| *Praomys misonnei* (F) | cytb (1105) | 54 (229, 120) | TMRCA in BEAST based on secondary calibrations | TMRCA clade IV | 0.32 | SR: RDC, North of the Congo River and East of Uganda |
|  |  |  |  | TMRCA clade III | 0.07 | SR: within Nigeria |
|  |  |  |  | TMRCA clade II | 0.42 | SR: Ghana and Benin, East of the Volta River (~Dahomey Gap) |
|  |  |  |  | TMRCA clade I+II+III | 0.64 | SR: three forest blocks between Dahomey Gap and ~Congo River |
|  |  |  |  | split clade II vs. III | 0.52 | CZ: SW Nigeriae, potentially Dahomey Gap |
|  |  |  |  | split clade IV vs. I+II+III | 0.90 | CZ: somewhere between Aruwimi and Oubangui Rivers |
| *Praomys rostratus* (F) | cytb (798) | 68 (352, 172) | TMRCA in BEAST based on *Mus/Rattus* divergence | TMRCA of clades I to XII | 0.89 to 0.10 | SR: restricted forest areas within the West African forest block |
|  |  |  |  | split clade I vs. II | 0.43 | CZ: overlap within Guinea |
|  |  |  |  | TMRCA of clades I+II+h26 | ~0.50 | SR: West African forest block |
|  |  |  |  | split clades III to VI vs. VI to XIII | 0.57 |  |
| *Praomys tullbergi* (F) | cytb (798) | 16 (107, 43) | TMRCA in BEAST based on *Mus/Rattus* divergence | TMRCA of the Eastern clade | 0.16 | SR: 'Baoulé V' triangle |
|  |  |  |  | split Eastern vs. Western clades | 0.31 | CZ: ~Comoé River |

* SOG, HZ, F and SSRS correspond to Savana and open grasslands, humid zone, forest and Saharo-Sahelian rocket and/or sandy habitats, respectively.

** Number of base pairs (for sequences) or loci (for microsatellites) is indicated between brackets.

*** Number of individuals and haplotypes are indicated between brackets.

**** Geographic aspects include hypothetic contact zones between lineages (CZ) or supposed spatial range of a given lineage (SR).
